# Anti-PD-1 Vasculitis of the central nervous system or radionecrosis?

**DOI:** 10.1186/s40425-017-0304-8

**Published:** 2017-12-19

**Authors:** Roger Sun, Francois-Xavier Danlos, Samy Ammari, Guillaume Louvel, Frédéric Dhermain, Stéphane Champiat, Olivier Lambotte, Eric Deutsch

**Affiliations:** 1 Department of Radiotherapy, Gustave Roussy Cancer Campus, 94800 Villejuif, France; 2 Immunity and Cancer Department, Institut Curie, PSL Research University, Institut National de la Santé et de la Recherche Médicale U932, 75005 Paris, France; 3 Department of Radiology, Gustave Roussy Cancer Campus, 94800 Villejuif, France; 4 Drug Development Department, Gustave Roussy Cancer Campus, 94800 Villejuif, France; 5 Department of Internal Medicine, Bicêtre Hopistal, 94270 Le Kremlin-Bicêtre, France; 6 Paris-Saclay University, 94270 Le Kremlin-Bicêtre, France

**Keywords:** Cerebral vasculitis, Immunotherapy, Radionecrosis

## Abstract

Commentary on « Cerebral vasculitis mimicking intracranial metastatic progression of lung cancer during PD-1 blockade » by Läubli H et al., J Immunother Cancer. 2017;5:46.

The authors diagnosed a cerebral tumor-like lymphocytic vasculitis associated with anti-endothelial cell auto-antibodies secondary to anti-PD-1 therapy, treated by surgical resection and corticosteroids. We thought that this diagnosis should be discussed for at least two reasons. First, etiological explorations were not sufficient. Second, the diagnostic of radionecrosis should also be discussed.

## Main text

In the June edition of Journal for ImmunoTherapy of Cancer, Läubli H et al. analyzed an interesting case report concerning a 53-year old man with stage IV non-squamous cell lung cancer with primary cerebral metastasis who developed an inflammatory tumor-like disease of the brain after anti PD-1 antibody treatment [1].

Primarily, the patient was treated with one fraction of 20 Gy stereotactic radiotherapy on the right parieto-temporal lesion and received four cycles of chemotherapy with cisplatin and pemetrexed. Two months later, he received nivolumab infusions, an anti PD-1 monoclonal antibody, as second line treatment for progressive disease. He rapidly developed focal neurological symptoms which seemed to be related to a new parieto-temporal lesion, suspected to be disease progression. Other lesions remained stable and he did not develop extra-neurological symptoms.

The parieto-temporal lesion was surgically removed and pathological analysis clearly eliminated a metastatic focus. The authors concluded a diagnosis of cerebral vasculitis, also called Angiitis of the Central Nervous System (ACNS). Indeed, histopathology seems to confirm necrotizing encephalitis associated with a perivascular infiltrate of T-cells. Although the patient had no preexisting auto-immune diseases, serum analysis revealed pre-existing anti-nuclear auto-antibody, anti-SSA/Ro and anti SSB/La antibodies. Moreover, at the time of the diagnosis of ACNS, serum anti-endothelial cells antibodies were observed, which prompted the authors to confirm this diagnosis.

ACNS is a rare and severe inflammatory disease of small leptomeningeal and parenchymal blood vessels of the brain, which could be caused by several neurologic insults such as malignancy, infection, ionizing radiation, and auto-immune disease [2]. Its main clinical manifestations are headache, focal neurological deficits, or seizures. Although tumor-like behavior is a well-known presentation of ACNS, they are more frequently observed as multifocal lesions of supratentorial cerebral ischemic infarctions, associated with hemorrhagic manifestations (parenchymal, subarachnoid or both) and leptomeningeal involvement in Magnetic Resonance Imaging (MRI) [3]. Pathological analysis of ACNS could show granulomatous, lymphocytic and/or necrotizing vasculitis [4]. The main lesions are perivascular, and extend through the vascular wall. Importantly, large and medium vessel involvement must be confirmed by cerebral conventional or magnetic resonance angiography. There are no specific auto-antibodies which permit confirmation of the diagnosis of ACNS.

In summary, the authors diagnosed a cerebral tumor-like lymphocytic vasculitis associated with anti-endothelial cell auto-antibodies secondary to anti-PD-1 therapy, treated by surgical resection and corticosteroids. We thought that this diagnosis should be discussed for at least two reasons.

First, if the patient had really developed cerebral vasculitis because of nivolumab, etiological explorations were not sufficient. The patient should have had a cerebral angiography, the gold standard in visualizing large or medium vessel arteritis to support diagnosis of ACNS, especially since there were no multifocal lesions, no signs of ischemic infarctions, no punctiform and linear enhancement, and no meningeal enhancement on the contrast-enhanced T1-weighted imaging. Further, no T2*-weighted or vascular sequences (TOF or Tricks) were performed to look for leptomeningeal hemorrhage or large vessel abnormalities. Moreover, although he did not develop systemic manifestations, a cryoglobulinemia associated vasculitis could be suspected by lymphocytic and necrotizing small vessels arteritis and presence of anti-nuclear, anti SSA and anti SSB antibodies [5]. Other systemic vasculitis should be eliminated, such as anti neutrophil cytoplasmic antibody (ANCA) associated vasculitis [6]. These points are important because primary and secondary angiitis of the central nervous system are severe diseases which should be treated with immunosuppressive drugs such as cyclophosphamide, mycophenolate mofetil or rituximab, and not only with surgical resection and corticosteroids [7]. The favorable outcome without any immunosuppressive treatment is also a strong argument against a primary or secondary cerebral vasculitis.

Second, the diagnostic of radionecrosis should also be discussed. Radionecrosis is the most common delayed complication of stereotactic radiotherapy in the treatment of brain metastases. Using the single high fraction dose radiotherapy used in this patient, radionecrosis occurs in about 10% of treated lesions, from 6 months to several years after treatment [8, 9]. Here, the timeline, the MRI findings, the response to corticotherapy and the localization of the new parieto-temporal lesion in the formerly irradiated area are all highly suggestive of radionecrosis. The imaging provided shows a nodular enhancement which is compatible with progression, but also with radionecrosis, corresponding to a leakage of gadolinium through the blood-brain barrier. A MR perfusion weighted imaging would then have shown changes in blood-brain barrier permeability and no increased relative cerebral blood volume (rCBV) [10]. Moreover, necrotizing and perivascular lymphocytic infiltrates found in the tissues are also compatible with this diagnosis, as shown in several pathological studies of radionecrosis [11]. The widely accepted pathophysiological hypothesis of radionecrosis is that direct primary injury to the blood vessels causes the brain parenchymal injury as secondary damage.

It is essential to note that the presence or development of auto anti-antibodies or anti-endothelial cell antibodies development do not confirm an auto-immune disease [12]. Clinical and/or radiological criteria are mandatory. In this case report in particular, the presence of anti-endothelial cell antibodies could be related to the radionecrosis leading to endothelial aggression. The specificity of anti-endothelial cell antibodies is not given and these antibodies could have targeted various antigens. Furthermore, it has been reported that patients who receive immunotherapy after stereotactic radiotherapy may have an increased rate of radionecrosis [13, 14].

For all these reasons, we think that “cerebral vasculitis” should not have been a definitive diagnosis for this patient. This very interesting case report highlights the need to establish strong collaborative networks between oncologists, radiotherapists, radiologists, organ specialists, internists and clinical immunologists. We do believe that the etiology of such rare events should be discussed and confronted in order to avoid misleading conclusions regarding the safety of immune checkpoint inhibitors and radiotherapy. Such combinations are currently the focus of more than 150 trials [15]. One should stress the absence of a straightforward randomized trial comparing stereotactic radiotherapy to stereotactic radiotherapy plus immunotherapy. It remains difficult to conclude the safety of the combination over irradiation alone which is known to give rise to 50% radionecrosis depending on the volume and dose-fractionation patterns [16]. It should however to be noted that a recent review of a large cohort of 85 patients receiving brain-directed radiation and anti-PD-1 did not report any radionecrosis, but favorable outcomes with prolonged survival [17].

Conclusions from limited size series always carry intrinsically their limits. For example, recent controversies related to the safety of BRAF inhibitors combined to brain radiotherapy with expediting adverse events have been solved by large enough series of data which eventually failed to confirm initial safety concerns [18]. Clinicians must disseminate toxicity data early on whenever possible. However, extreme caution should be standard to avoid the confusion between small and larger series of patients.

## Abbreviations

ACNS: Angiitis of the Central Nervous System
ANCA: Anti neutrophil cytoplasmic antibody
MRI: Magnetic Resonance Imaging
PD-1: Programmed cell death 1
rCBV: Relative cerebral blood volume

## References

[1] Läubli H, Hench J, Stanczak M, Heijnen I, Papachristofilou A, Frank S (2017). Cerebral vasculitis mimicking intracranial metastatic progression of lung cancer during PD-1 blockade. J Immunother Cancer.

[2] de Boysson H, Zuber M, Naggara O, Neau J-P, Gray F, Bousser M-G, et al. Primary angiitis of the central nervous system: description of the first fifty-two adults enrolled in the French cohort of patients with primary vasculitis of the central nervous system. Arthritis Rheumatol Hoboken NJ. mai 2014;66(5):1315–1326. 10.1002/art.38340 24782189

[3] Boulouis G, de Boysson H, Zuber M, Guillevin L, Meary E, Costalat V, et al. Primary Angiitis of the central nervous system: magnetic resonance imaging Spectrum of Parenchymal, Meningeal, and vascular lesions at baseline. Stroke. mai 2017;48(5):1248–1255. 10.1161/STROKEAHA.116.016194 28330942

[4] Miller DV, Salvarani C, Hunder GG, Brown RD, Parisi JE, Christianson TJ, et al. Biopsy findings in primary angiitis of the central nervous system. Am J Surg Pathol. janv 2009;33(1):35–43. 10.1097/PAS.0b013e318181e097 18941399

[5] Lioger B, Ferreira-Maldent N, Cottier JP, Debiais S, Gyan E, Maillot F. Rituximab for Sjögren syndrome-associated type II mixed cryoglobulinemic cerebral vasculitis. Neurol Neuroimmunol Neuroinflammation. août 2016;3(4):e253. 10.1212/NXI.0000000000000253 PMC4949984 27458597

[6] De Luna G, Terrier B, Kaminsky P, Le Quellec A, Maurier F, Solans R, et al. Central nervous system involvement of granulomatosis with polyangiitis: clinical-radiological presentation distinguishes different outcomes. Rheumatol Oxf Engl. mars 2015;54(3):424–432. 10.1093/rheumatology/keu336 25187644

[7] Vera-Lastra O, Sepúlveda-Delgado J, Cruz-Domínguez Mdel P, Medina G, Casarrubias-Ramírez M, Molina-Carrión LE, et al. Primary and secondary central nervous system vasculitis: clinical manifestations, laboratory findings, neuroimaging, and treatment analysis. Clin Rheumatol. avr 2015;34(4):729–738. 10.1007/s10067-014-2831-8 25425493

[8] Shaw E, Scott C, Souhami L, Dinapoli R, Kline R, Loeffler J, et al. Single dose radiosurgical treatment of recurrent previously irradiated primary brain tumors and brain metastases: final report of RTOG protocol 90-05. Int J Radiat Oncol. mai 2000;47(2):291–298. 10.1016/s0360-3016(99)00507-6 10802351

[9] Blonigen BJ, Steinmetz RD, Levin L, Lamba MA, Warnick RE, Breneman JC. Irradiated volume as a predictor of brain radionecrosis after linear accelerator stereotactic radiosurgery. Int J Radiat Oncol Biol Phys. 15 juill 2010;77(4):996–1001. 10.1016/j.ijrobp.2009.06.006 19783374

[10] Shah R, Vattoth S, Jacob R, Manzil FFP, O’Malley JP, Borghei P, et al. Radiation necrosis in the brain: imaging features and differentiation from tumor recurrence. Radiographics sept 2012;32(5):1343–1359. 10.1148/rg.325125002 22977022

[11] Yoshii Y (2008). Pathological review of late cerebral radionecrosis. Brain Tumor Pathol.

[12] Legendre P, Régent A, Thiebault M, Mouthon L. Anti-endothelial cell antibodies in vasculitis: a systematic review. Autoimmun Rev. févr 2017;16(2):146–153. 10.1016/j.autrev.2016.12.012 27989761

[13] Colaco RJ, Martin P, Kluger HM, Yu JB, Chiang VL. Does immunotherapy increase the rate of radiation necrosis after radiosurgical treatment of brain metastases? J Neurosurg. juill 2016;125(1):17–23. 10.3171/2015.6.JNS142763 26544782

[14] Du Four S, Wilgenhof S, Duerinck J, Michotte A, Binst AV, Ridder MD, et al. Radiation necrosis of the brain in melanoma patients successfully treated with ipilimumab, three case studies. Eur J Cancer. 1 nov 2012;48(16):3045–3051. 10.1016/j.ejca.2012.05.016 22727601

[15] Search of: immunotherapy radiotherapy | Recruiting, Active, not recruiting, Enrolling by invitation Studies - List Results - ClinicalTrials.gov [Internet]. [cité 7 juill 2017]. Disponible sur: https://clinicaltrials.gov/ct2/results?term=immunotherapy+radiotherapy&Search=Apply&recrs=a&recrs=f&recrs=d&age_v=&gndr=&type=&rslt=.

[16] Rhun EL, Dhermain F, Vogin G, Reyns N, Metellus P. Radionecrosis after stereotactic radiotherapy for brain metastases. Expert Rev Neurother. 2 août 2016;16(8):903–914. 10.1080/14737175.2016.1184572 27177183

[17] Pike LRG, Bang A, Ott P, Balboni T, Taylor A, Catalano P, et al. Radiation and PD-1 inhibition: Favorable outcomes after brain-directed radiation. Radiother Oncol. 2017;124:98–103. 10.1016/j.radonc.2017.06.006 28662869

[18] Anker CJ, Grossmann KF, Atkins MB, Suneja G, Tarhini AA, Kirkwood JM. Avoiding severe toxicity from combined BRAF inhibitor and radiation treatment: consensus guidelines from the eastern cooperative oncology group (ECOG). Int J Radiat Oncol Biol Phys. 1 juin 2016;95(2):632–646. 10.1016/j.ijrobp.2016.01.038 PMC5102246 27131079

